# A Systematic Review of Length of Stay Linked to Hospital-Acquired Falls, Pressure Ulcers, Central Line–Associated Bloodstream Infections, and Surgical Site Infections

**DOI:** 10.1016/j.mayocpiqo.2025.100607

**Published:** 2025-04-08

**Authors:** Bashar Hasan, Dima Bechenati, Hannah M. Bethel, Sydney Cho, Noora S. Rajjoub, Sammy T. Murad, Adel Kabbara Allababidi, Tamim I. Rajjo, Mohammed Yousufuddin

**Affiliations:** a Evidence-Based Practice Center, Kern Center for the Science of Healthcare Delivery, Mayo Clinic, Rochester, MN; b Division of Public Health, Infectious Diseases and Occupational Medicine, Mayo Clinic, Rochester, MN; c Department of Family Medicine, Mayo Clinic, Rochester, MN; d Department of Hospital Internal Medicine, Mayo Clinic Health System, Austin, MN

## Abstract

**Objective:**

To systematically review hospital length of stay (LOS) associated with falls, pressure ulcers, central line–associated bloodstream infections, and surgical site infections and their potential differences before and after the implementation of the hospital-acquired condition (HAC) reduction program (HACRP).

**Methods:**

We searched PubMed, Embase, and Cochrane databases from January 1, 2000, to May 26, 2024, for studies examining LOS and health care costs in patients with any of the 4 HACs. Studies included prospective and retrospective cohorts and case-control designs across various clinical settings.

**Results:**

Fifty studies involving 45,080,370 patients admitted for medical and surgical conditions met the inclusion criteria, with 1,939,151 patients experiencing 1 or more HACs. Length of stay increased by an average of 5.2 days for falls, 12.9 days for pressure ulcers, 22.1 days for central line–associated bloodstream infections, and 7.9 days for surgical site infections. After HACRP implementation, LOS for falls increased by 4.9 days, whereas LOS for pressure ulcers decreased by 39.1 days.

**Conclusion:**

This systematic review presents detailed data on excess LOS for 4 selected HACs across medical, surgical, intensive care unit, and rehabilitation settings over the past 25 years. The varying impact of HACRP on different HACs highlights the need for targeted prevention strategies.

Each year, millions of patients worldwide, including those in the United States, experience adverse events of varying severity during hospitalization, ranging in severity and posing substantial public health concerns.^1^, ^2^, ^3^ Many of these acute conditions, unrelated to the patient’s primary diagnosis, have been recognized as a major concern by the US Centers for Medicare and Medicaid Services (CMS). To address this, CMS has classified 14 categories of hospital-acquired conditions (HACs) for public reporting, with the goal of increasing transparency and accountability in health care.^4^

Decreasing mortality and morbidity associated with HACs has become a national priority in the United States.^5^ Despite federal evidence-based guidelines and policies designed to improve injury prevention and infection control, HACs remain common in hospitals, contributing markedly to morbidity and mortality.^1^^,^^6^^,^^7^ HACs not only harm patients but also erode public trust, affect hospital reimbursement rates, and damage institutional reputations.^8^^,^^9^ Consequently, CMS and the Agency for Healthcare Research and Quality (AHRQ) have led initiatives to reduce HAC rates and improve patient safety.^10^, ^11^, ^12^ In 2017, AHRQ released an evidence-based report outlining the additional hospital inpatient costs and mortality associated with 10 selected HACs.^13^

Many HACs are preventable through the application of best practices and evidence-based interventions.^14^^,^^15^ A thorough understanding of the underlying issues, combined with accurate measurement, is essential for effective prevention. Hospital length of stay (LOS) associated with HACs currently place a substantial burden on hospital resources and finances. However, these factors are potentially modifiable. A comprehensive meta-analysis supported by AHRQ examined the LOS and health care costs associated with 10 selected HACs using studies conducted before 2018.^13^ Although these analyses provide valuable insights into the burden these conditions place on US hospitals, there are limited recent data and comparisons between earlier and more contemporary studies. Reliable estimates of LOS are crucial for assessing the clinical, economic, and operational impacts of HAC-prevention strategies.

These 4 HACs—falls, pressure ulcers, central line–associated bloodstream infections (CLABSI), and surgical site infections (SSIs)—were selected because of their substantial impact on health care costs and patient outcomes. For example, CLABSIs can increase LOS by an average of 19 days, with costs reaching up to $90,000 per case.^16^, ^17^, ^18^ Similarly, SSIs are associated with longer hospital stays and can result in costs exceeding $40,000 per case.^16^^,^^19^^,^^20^ Pressure ulcers and falls, meanwhile, can lead to costs surpassing $15,000 per case.^16^ These conditions are among the most common and costly HACs, markedly contributing to patient morbidity, mortality, health care expenditures, and hospital workload. They are also publicly reported by the CMS and are primary targets for national patient safety initiatives aimed at reducing HAC rates and associated costs.^4^^,^^16^^,^^21^

To further explore the excess LOS, an indicator of hospital efficiency, associated with 4 selected HACs, we conducted a systematic review to summarize existing evidence and synthesize data from individual studies spanning 25 years. Our primary goal was to provide updated data on LOS for each of the selected HACs. Additionally, we sought to compare LOS across these conditions and assess potential differences between studies conducted before and after the implementation of the CMS-sponsored hospital-acquired condition reduction program (HACRP) in 2014.^6^ This analysis aimed to evaluate the program’s impact in reducing hospital resource burdens associated with these conditions. Although cost outcomes were also included in our systematic review design and protocol, variations in reporting and the inability to adjust for inflation across studies precluded direct comparisons. Therefore, cost data are presented in the Supplemental Material (available online at http://www.mcpiqojournal.org).

## Methods

This systematic review followed the Preferred Reporting Items for Systematic reviews and Meta-Analyses guidelines for systematic reviews and meta-analyses.^22^ The study adhered to the synthesis without meta-analysis statement.^23^

### Objectives

We conducted a systematic review to evaluate LOS and direct health care costs associated with 4 HACs: falls, pressure ulcers, CLABSIs, and SSIs. A secondary aim was to assess differences in LOS and costs before and after HACRP implementation in 2014.

### Outcomes

The 2 primary outcomes were LOS and direct health care costs for the 4 selected HACs. If both outcomes were available, studies were included in both categories. Another outcome was LOS and health care costs, stratified by periods 2000-2013 and 2014-2024.

### Eligibility

We included studies on LOS or inpatient health care costs associated with the selected HACs. These studies covered various elective and emergency medical or surgical conditions, including intensive care unit (ICU) and rehabilitation settings. We considered retrospective and prospective observational studies, cross-sectional studies, and case-control designs from single or multiple centers, as well as nationwide data sets. Comparators included patients admitted without any specified HACs.

### Search Strategy and Selection of Studies

We conducted a comprehensive search of several databases from 2000 to May 29, 2024, in English. Databases included Ovid MEDLINE, EMBASE, Cochrane Central, Cochrane Database of Systematic Reviews, and Scopus. The search strategy, developed by an experienced librarian and the principal investigator, used controlled vocabulary and keywords related to falls, pressure ulcers, CLABSI, and SSIs. The search was conducted using the following terms: *fall, surgical site infection, pressure ulcer, catheter-related infection, length of stay, hospital costs, and healthcare expenditures*. The complete search strategy is in the Supplementary Material.

### Selection of Included Studies

Pairs of investigators (B.H., D.B., H.M.B., S.C., N.S.R., S.T.M., A.K., and T.I.R.) independently screened citations and reviewed selected articles. Discrepancies were resolved by a third reviewer (B.H. or M.Y.), acting as an arbitrator. Citations were imported into EndNote 21 (Clarivate Analytics) to remove duplicates. Titles and abstracts were screened for eligibility based on predefined criteria, using DistillerSR software (DistillerSR Inc) to organize the process. Full-text reviews determined eligibility for final inclusion.

### Data Extraction

We extracted the following data, detailed in Supplemental Table 1 (available online at http://www.mcpiqojournal.org): study year, design, country, setting (eg, single center and academic hospital), admission diagnosis, and population characteristics (age, sex, and group sizes). We also captured the specific HAC, LOS, and health care costs, organizing all data in an Excel sheet.

### Methodologic Quality Assessment

We assessed study quality using an adapted Newcastle-Ottawa Scale (NOS).^24^ Each study’s bias risk was categorized as low, moderate, or high (Supplemental Table 2, available online at http://www.mcpiqojournal.org).

### Data Synthesis

Hospital settings were grouped as general inpatient, ICU, and rehabilitation. Falls were classified into categories based on injury severity. Surgical site infections were divided into subgroups (eg, superficial, deep, and organ space) and surgical type.

We pursued a thematic narrative synthesis and presented descriptive statistics because data were heterogeneous and not amenable to meta-analysis. We stratified findings based on publication date, before and after the implementation of the HACRP in 2014 to allow comparative analysis of potential shifts in LOS and health care cost. Data analysis and figure creation were done using Python in Anaconda (Jupyter Notebook, version 7.0.8), with pandas for data manipulation, matplotlib for plots, and seaborn for heatmaps. We visualized mean differences in LOS and incremental costs across the 4 HACs.

**Figure:**
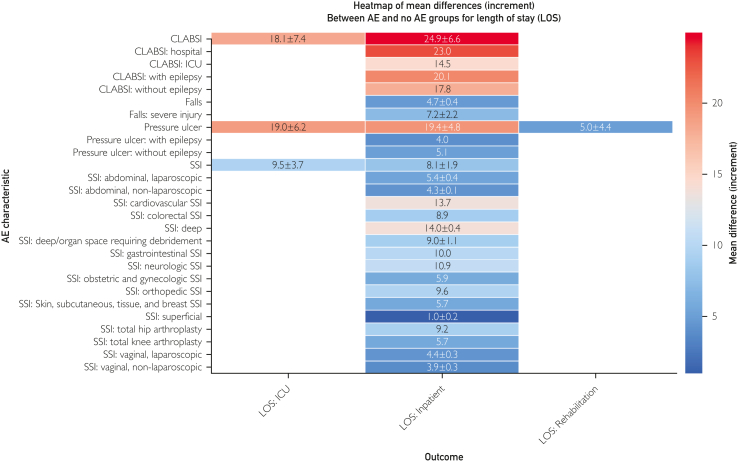
Heatmap of mean differences in length of stay (LOS) for selected hospital-acquired conditions (HACs) between adverse event (AE) and no AE groups across ICU, general inpatient, and rehabilitation settings. CLABSI, central line–associated bloodstream infection; ICU, intensive care unit; SSI, surgical site infection.

## Results

### Characteristics of Included Studies

The search yielded 5548 records, of which 155 full-text articles were reviewed. Ultimately, 63 reports met the criteria for LOS evaluation and 61 for cost analysis, covering the 4 HACs from 50 unique studies (Supplemental Box, available online at http://www.mcpiqojournal.org). Supplemental Figure 1 (available online at http://www.mcpiqojournal.org) is a Preferred Reporting Items for Systematic reviews and Meta-Analyses flow diagram of the selection process. All studies included in this review were conducted in the United States and published between 2001 and 2023. Studies from non-US countries were excluded to maintain consistency in cost estimates, as variations in health care systems, resource utilization, reimbursement models, and economic factors can considerably influence cost calculations and LOS estimates. Thirty-five studies were based on nationwide data, spanning diverse regions and health care settings. Each outcome category (LOS and cost) for the 4 selected HACs—falls, pressure ulcers, CLABSI, and SSIs—was divided into 2 timeframes for analysis (2000-2013 and 2014-2024) as illustrated in the Table for LOS and in Supplemental Table 3 (available online at http://www.mcpiqojournal.org) for cost. In total, these studies included 45,080,370 patients, with 1,939,151 patients experiencing 1 or more HACs. The remaining 43,141,219 patients did not experience HACs. The characteristics of the included studies are presented in Supplemental Table 1.

**Table:** Comparison of Length of Stay Differences for Selected HACs Before and After HACRP Implementation (2000-2024)

HAC	2000-2024 studies			2000-2013 studies, pre-HACRP			2014-2024 studies, post-HACRP			Difference in mean value
	No. studies	No. patients	Mean excess value	No. studies	No. patients	Mean excess value	No. studies	No. patients	Mean excess value	
Falls	5	1,130,257	4.7	2	1,088,168	1.0	3	42,089	5.9	4.9 ↑
Pressure ulcer	10	6,605,318	19.4	2	1,943,673	42.9	8	4,655,841	3.8	39.1↓
CLABSI	16	24,387	24.9	4	2824	26.0	12	21,563	24.6	1.4↓
SSI	32	6,016,548	8.1	15	1,942,717	10.3	17	4,073,831	7.2	3.1↓

Length of stay (LOS): For falls, the average hospital stay increased post-HACRP, with a mean excess of 5.9 days in the 2014-2024 studies, compared with just 1.0 day in the pre-HACRP studies from 2000 to 2013, resulting in a 4.9-day increase in LOS. In the case of pressure ulcers, LOS considerably decreased post-HACRP, dropping from 42.9 days in the pre-HACRP period to 3.8 days post-HACRP, a reduction of 39.1 days. For CLABSI, the change in LOS was minimal, with a slight reduction of 1.4 days post-HACRP, from 26.0 days pre-HACRP to 24.6 days afterward. Similarly, for SSI, the LOS decreased by 3.1 days, from 10.3 days pre-HACRP to 7.2 days post-HACRP.

CLABSI, central line–associated bloodstream infection; HAC, hospital-acquired condition; HACRP, hospital-acquired condition reduction program; ICU, intensive care unit; SSI, surgical site infection.

Of the LOS studies from 2000 to 2024, 5 focused on falls (1,130,257 patients), 10 on pressure ulcers (6,605,318 patients), 16 on CLABSI (24,387 patients), and 32 on SSIs (6,016,548 patients). Similarly, in cost-evaluating studies from 2014 to 2024, 10 focused on falls (1,184,802 patients), 6 on pressure ulcers (4,639,644 patients), 12 on CLABSI (94,802 patients), and 33 on SSIs (25,432,527).

### Length of Hospital Stay

#### Falls

Five studies from 2000 to 2024 reported an average excess LOS of 4.7 days for patients who experienced a fall. Among these, 2 studies published before 2014 with 1,088,168 patients reported a mean excess LOS of 1.0 day, whereas 3 studies from 2014 onward with 42,089 patients indicated a larger increase of 5.9 days for those with falls.

#### Pressure Ulcers

Ten studies from 2000 to 2024 reported an average excess LOS of 19.4 days for patients with pressure ulcers compared with that for those without. Of these, 2 studies published from 2000 to 2013 with 1,943,673 patients reported a larger excess LOS of 42.9 days, and 8 studies since 2014 with 4,655,841 patients reported a smaller excess LOS of 3.8 days for patients with pressure ulcers compared with that for those without.

#### Central Line–Associated Bloodstream Infections

Sixteen studies from 2000 to 2024 estimated a mean excess LOS of 24.9 days for patients with CLABSI compared with that for those without. Of these, 4 studies (2000-2013) with 2824 patients reported a mean excess LOS of 26.0 days and 12 studies (2014-2024) with 21,563 patients recorded a slightly lower mean excess LOS of 24.6 days.

#### Surgical Site Infections

Thirty-two studies from 2000 to2024 reported a mean excess LOS of 8.1 days for patients with SSIs compared with that for those without. Of these, 15 studies published from 2000 to 2013 with 1,942,717 patients reported a LOS increase of 10.3 days, whereas 17 studies since 2014 with 4,073,831 patients reported a smaller LOS increase of 7.2 days.

### Comparative Analysis

The Figure presents a heat map of mean LOS differences between patients with and without the 4 selected HACs across ICU, general inpatient, and rehabilitation settings. Among conditions, CLABSI reported the largest LOS increase: 24.9 days in general inpatient and 18.1 days in ICU settings. Pressure ulcers considerably prolonged LOS by 19.4 days in general inpatient, 19.0 days in ICU, and 5.0 days in rehabilitation. Falls increased LOS by 4.7 days, with severe injuries extending it by 7.2 days. Surgical site infections were classified as superficial or deep and further categorized by organ system to assess LOS impact. On average, SSIs increased LOS by 8.1 days in general inpatient and 9.5 days in ICU. Deep SSIs were associated with a 14.0-day increase, with cardiovascular operations contributing the most prolonged stays at 13.7 days. Supplemental Figure 2 (available online at http://www.mcpiqojournal.org) illustrates the average incremental health care costs for each HAC.

## Discussion

To our knowledge, this is the first systematic review since the AHRQ report, which was based on data collected up to and including 2017. Our review extends this analysis by covering both earlier and more recent studies, published between 2000 and 2024, spanning approximately 25 years of research and including data from 45,080,370 patients. On the basis of this extensive review, we identified 4 key findings.

First, the average excess LOS for the 4 selected HACs varied almost 25-fold, depending on the type of HAC, the specific study, and its publication timeframe. The excess mean LOS ranged from 1.0 day for patients with simple superficial SSIs to 24.9 days for patients with more complex CLABSI, compared with that for patients without HACs, as observed across all 63 studies. Second, when comparing LOS by timeframes, we found that the excess LOS for falls increased, whereas it decreased for pressure ulcers, CLABSI, and SSIs between the periods 2000-2013 and 2014-2024. This shift coincides with the implementation of the HACRP in 2014, which was aimed to incentivize hospitals to improve patient safety and reduce the incidence of HACs.^6^ These findings underscore the varying LOS trends for the selected HACs over time, highlighting the need for continued efforts to optimize hospital resource utilization and patient safety initiatives. From the time periods 2000-2013 to 2014-2024, excess LOS for falls increased, whereas it decreased for pressure ulcers, CLABSI, and SSIs.

Decreasing mortality and morbidity associated with HACs remains a national priority in the United State.^5^ Despite various federal initiatives, including penalties for hospitals with HAC rates above the national average, progress has been slow. Evidence also suggests that the implementation of the HACRP in 2014 had only a limited impact on reducing HAC-related morbidity and mortality.^25^ Contributing factors may include the growing complexity of health care delivery, inconsistent adherence to best practices, and the need for more comprehensive strategies. Ongoing evaluation and refinement of the HACRP will be essential to achieve its intended objectives. Many of these HACs are preventable^26^, ^27^, ^28^ and contribute to prolonged hospital stays.

The systematic review spans a 25-year period, encompassing large-scale studies published between 2000 and 2024, providing a broad perspective in LOS for 4 selected HACs. In this systematic review, we prioritized LOS as the primary outcome, whereas cost data were included as a secondary outcome (Supplemental Material). A key limitation of cost findings is the lack of inflation adjustments across studies. Because reliable cost comparisons require inflation-adjusted estimates derived from full original data sets, which were unavailable, direct cost comparisons over time may be less reliable. Therefore, although cost estimates provide useful context, LOS remains the most robust and generalizable measure of the burden associated with these HACs.

This study highlighted evolving hospital resource utilization over time. The review captured a range of health care settings such as general medical-surgical units, ICUs, and rehabilitation facilities, providing a more generalizable understanding of LOS impacts for 4 selected HACs in real-world patient population. The subgroup analyses of falls and SSIs further strengthens the conclusions.

The study results should be interpreted cautiously in the following context. First, although we used the NOS, a widely recognized robust tool for assessing the quality of observational studies, it has limitations when applied to time-dependent events such as HACs. The NOS does not account for the temporal nature of HACs or the complexities of attributing outcomes, such as LOS or costs, which could result in biased estimates if appropriate statistical methods are not used.^29^^,^^30^ Second, the LOS estimates for CLABSI and SSI were unexpectedly large. On revisiting the included studies, we confirmed the accuracy of the reported data but attribute these findings to variations in study methodologies, differences in patient populations, the presence of outliers, or the specific contexts and settings of the studies. Third, the included studies did not provide stratified data on different stages of pressure ulcers (stages 1 to 4). This lack of granularity led to lumped estimates that likely introduce bias because stages 1 and 2 typically have minimal impact on LOS, whereas stages 3 and 4 are rare but substantially prolong hospitalization. Fourth, HACs are time-dependent events, but many included studies did not account for this variability in their methodologies. This oversight may contribute to biased estimates and underscores the importance of using appropriate time-dependent statistical methods in future research. Additional limitations were that the included studies were heterogeneous in design, methods, and reporting, limiting the ability to conduct a meta-analysis or draw comprehensive conclusions on how LOS varies across different settings and conditions. Some of the included studies may have overlapping data, which could introduce bias and affect the overall findings. The review analyzed 4 of 14 HACs specified by the CMS, leaving gaps in understanding the full spectrum of HAC-related costs and resource use. Although the estimates for falls, pressure ulcers, CLABSI, and SSIs are based on reportable events to CMS and derived from standardized methodologies, variability in coding practices and differences in health care settings may impact their generalizability. Additionally, although these estimates are considered reliable owing to regulatory oversight, further validation in diverse populations and settings would strengthen their applicability.

## Conclusion

This systematic review provides detailed data on excess LOS for 4 selected HACs across various medical, surgical, ICU, and rehabilitation settings over the past 25 years. Excess LOS ranged from 1.0 day for superficial SSIs to 24.9 days for complex CLABSI. From 2000-2013 to 2014-2024, LOS decreased for pressure ulcers, CLABSI, and SSIs, whereas it increased for falls. However, the findings should be interpreted cautiously owing to key limitations, including methodologic differences, data constraints, and challenges in attributing LOS outcomes to health care–acquired conditions.

## Potential Competing Interests

The authors report no competing interests.

## Ethics Statement

Not applicable.
