# Supplementary material for: A Systematic Review of Length of Stay Linked to Hospital-Acquired Falls, Pressure Ulcers, Central Line–Associated Bloodstream Infections, and Surgical Site Infections
Source: Mayo Clin Proc Innov Qual Outcomes. 2025 Apr 8;9(3):100607. doi: 10.1016/j.mayocpiqo.2025.100607 (PMC12008517; doi:10.1016/j.mayocpiqo.2025.100607)
Supplement: Supplemental Material [file mmc1.pdf]

Supplemental Material

A Systematic Review of Length of Stay Linked to Hospital-Acquired Falls, Pressure Ulcers, CLABSI, and Surgical Site Infections

Bashar Hasan, Dima Bechenati, Hannah M. Bethel, Sydney Cho, Noora S. Rajjoub, Sammy Murad, Adel Kabbara Allababidi, Tamim I. Rajjo, Mohammed Yousufuddin

Page

1. Search Strategy…………………………………………………………………...…2
2. Figure 1. PRISMA Flow Diagram……………………………………..………...5
3. Figure 2. Average incremental cost for each adverse event……………………………6
4. Box 1. Bibliography of Included Studies…………………………………...………7
5. Table 1. Study Characteristics……………………………….…………………11
6. Table 2. Methodological Quality……………………………………………………..19
7. Table 3. Comparison of cost differences before and after HACRP Implementation...22
8. Table 4. PRISMA Check List………………………………………..................23

Search strategy

Ovid

Database(s): EBM Reviews - Cochrane Central Register of Controlled Trials April 2024, EBM Reviews - Cochrane Database of Systematic Reviews 2005 to May 22, 2024, Embase 1974 to 2024 May 24, Ovid MEDLINE(R) and Epub Ahead of Print, In-Process, In-Data-Review & Other Non-Indexed Citations, Daily and Versions 1946 to May 24, 2024

Search Strategy:

#	Searches	Results
1	exp Accidental Falls/	83179
2	(fall or falling or falls).ti,ab,kf.	511819
3	exp Surgical Wound Infection/	112126
4	((postop* or "post-op*" or surg* or resect* or operat* or repair* or reconstruction* or excis*) and infect*).ti,ab,kf.	723259
5	exp Pressure Ulcer/	41953
6	("bed sore" or "bed sores" or "decubitus sore" or "decubitus sores" or "decubitus ulcer" or "decubitus ulcers" or "pressure injuries" or "pressure injury" or "pressure sore" or "pressure sores" or "pressure ulcer" or "pressure ulcers").ti,ab,kf.	38673
7	exp Catheter-Related Infections/ and ("central line*" or "central venous catheter*").ti,ab,kf.	9645
8	((("central line*" or "central venous catheter*") and infect*) or CLABSI* or CLBSI*).ti,ab,kf.	24394
9	or/1-8	1368457
10	exp Health Care Costs/	431030
11	exp "Costs and Cost Analysis"/	705944
12	exp Health Expenditures/	380284
13	exp Economics/	945544
14	exp "Cost of Illness"/	57342
15	exp Hospital Costs/	59738
16	exp Cost-Benefit Analysis/	203163
17	exp Economics, Hospital/	1101317
18	economics.fs.	460461
19	exp "Length of Stay"/	403861
20	(((length* or hospital*) adj3 stay*) or (recover* adj3 time*) or cost or costs or economic* or expenditure*).ti,ab,kf.	3222356
21	or/10-20	4627424
22	9 and 21	181570
23	exp Inpatients/	281441

24	("Burn Unit*" or "Coronary Care Unit*" or hospital* or ICU or inhospital* or inpatient* or "in-patient*" or "intensive care" or "operating room*" or "Recovery Room*" or "Respiratory Care Unit*" or ward or wards).ti,ab,kf.	10359340
25	23 or 24	10376638
26	22 and 25	114898
27	exp United States/	2915444
28	(alabama or alaska or "appalachian region" or arizona or arkansas or baltimore or boston or california or chicago or colorado or "confederate states of america" or connecticut or delaware or "district of columbia" or florida or "Georgia us" or "great lakes region" or hawaii or idaho or illinois or indiana or iowa or kansas or kentucky or "los angeles" or louisiana or maine or maryland or massachusetts or michigan or "mid-atlantic region" or minnesota or mississippi or missouri or montana or nebraska or nevada or "new england" or "new hampshire" or "new jersey" or "new mexico" or "New Orleans" or "new york" or "north carolina" or "north dakota" or ohio or oklahoma or oregon or "pacific states" or pennsylvania or philadelphia or "rhode island" or "san francisco" or "south carolina" or "south dakota" or tennessee or texas or "United States" or usa or utah or vermont or virginia or washington or "west virginia" or wisconsin or wyoming).ti,ab,kf.	2305864
29	27 or 28	4166411
30	26 and 29	11450
31	limit 30 to english language [Limit not valid in CDSR; records were retained]	11329
32	limit 30 to no language specified [Limit not valid in CDSR; records were retained]	17
33	31 or 32	11330
34	limit 33 to yr="2000 -Current"	9991
35	limit 34 to (conference abstract or editorial or erratum or note or addresses or autobiography or bibliography or biography or blogs or comment or dictionary or directory or interactive tutorial or interview or lectures or legal cases or legislation or news or newspaper article or overall or patient education handout or periodical index or portraits or published erratum or video-audio media or webcasts or clinical trial protocol) [Limit not valid in CCTR,CDSR,Embase,Ovid MEDLINE(R); records were retained]	2223
36	from 35 keep 226-234	9
37	(34 not 35) or 36	7777
38	limit 37 to yr="2020 -Current"	2198
39	remove duplicates from 38	1563
40	37 not 38	5579
41	remove duplicates from 40	3929
42	39 or 41	5492

Scopus

1 TITLE-ABS-KEY(fall or falling or falls)
2 TITLE-ABS-KEY(((postop* or "post-op*" or surg* or resect* or operat* or repair* or reconstruction* or excis*) and infect*))
3 TITLE-ABS-KEY("bed sore" OR "bed sores" OR "decubitus sore" OR "decubitus sores" OR "decubitus ulcer" OR "decubitus ulcers" OR "pressure injuries" OR "pressure injury" OR "pressure sore" OR "pressure sores" OR "pressure ulcer" OR "pressure ulcers")
4 TITLE-ABS-KEY((("central line*" or "central venous catheter*") and infect*) OR CLABSI* OR CLBSI*)
5 1 or 2 or 3 or 4
6 TITLE-ABS-KEY(((length* or hospital*) W/3 stay*) OR (recover* W/3 time*) OR cost OR costs OR economic* OR expenditure*)
7 TITLE-ABS-KEY("Burn Unit*" OR "Coronary Care Unit*" OR hospital* OR ICU OR inhospital* OR inpatient* OR "in-patient*" OR "intensive care" OR "operating room*" OR "Recovery Room*" OR "Respiratory Care Unit*" OR ward OR wards)
8 TITLE-ABS-KEY(alabama or alaska or "appalachian region" or arizona or arkansas or baltimore or boston or california or chicago or colorado or "confederate states of america" or connecticut or delaware or "district of columbia" or florida or "Georgia us" or "great lakes region" or hawaii or idaho or illinois or indiana or iowa or kansas or kentucky or "los angeles" or louisiana or maine or maryland or massachusetts or michigan or "mid-atlantic region" or minnesota or mississippi or missouri or montana or nebraska or nevada or "new england" or "new hampshire" or "new jersey" or "new mexico" or "New Orleans" or "new york" or "north carolina" or "north dakota" or ohio or oklahoma or oregon or "pacific states" or pennsylvania or philadelphia or "rhode island" or "san francisco" or "south carolina" or "south dakota" or tennessee or texas or "United States" or usa or utah or vermont or virginia or washington or "west virginia" or wisconsin or wyoming)
9 PUBYEAR AFT 1999 AND LANGUAGE(english)
10 5 and 6 and 7 and 8 and 9
11 DOCTYPE(ab) OR DOCTYPE(ed) OR DOCTYPE(bk) OR DOCTYPE(er) OR DOCTYPE(no) OR DOCTYPE(sh)
12 10 and not 11
13 INDEX(embase) OR INDEX(medline) OR PMID(0* OR 1* OR 2* OR 3* OR 4* OR 5* OR 6* OR 7* OR 8* OR 9*)
14 12 and not 13

Supplemental Figure 1: PRISMA Flow Diagram of Study Selection

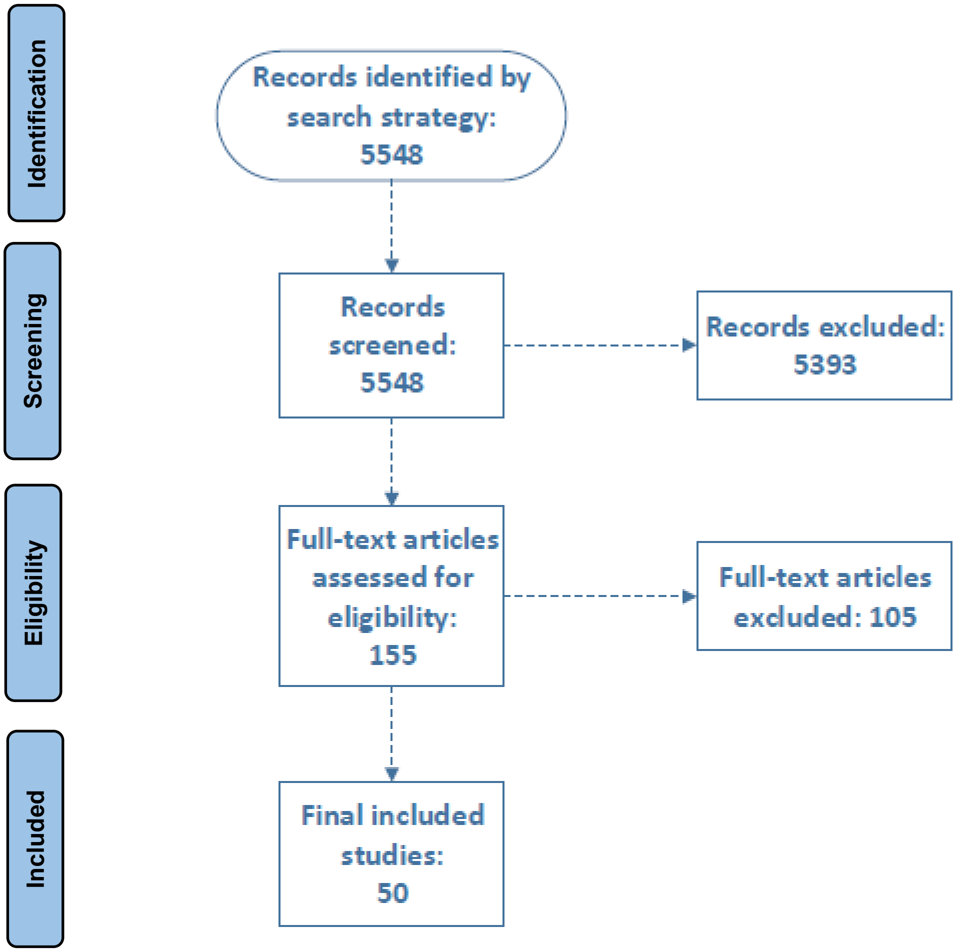

Abbreviations: PRISMA, Preferred Reporting Items for Systematic reviews and Meta-Analyses.

Supplemental Figure 2: Average incremental cost for each adverse event

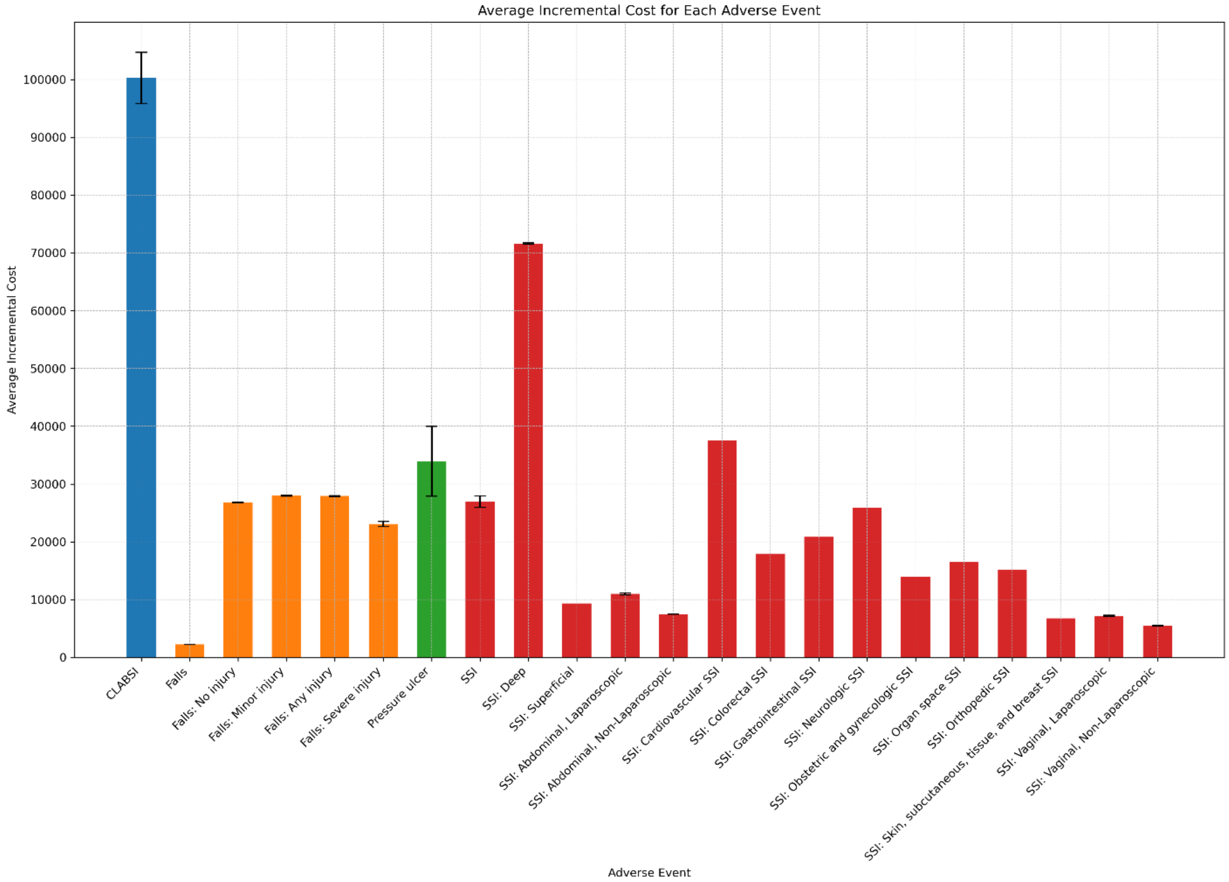

Supplemental Box: Bibliography of included studies

1. Yin C, Mpofu E, Brock K, Li X, Zhan R. Sacral Ulcer Development Risk Among Older Adult Patients in North Texas Rehabilitation Hospitals: Role of Comorbidities, Lifestyle, and Personal Factors. *J Gerontol Nurs*. 2024;50:32-41. doi: 10.3928/00989134-20240110-05
2. Lu W, Bloom O, Rathgeber M, Maltser S. Pressure injury prevalence and characteristics in patients with COVID-19 admitted to acute inpatient rehabilitation unit. *Frontiers in Rehabilitation Sciences*. 2023;4:1058982. doi: https://dx.doi.org/10.3389/fresc.2023.1058982
3. Dykes PC, Curtin-Bowen M, Lipsitz S, Franz C, Adelman J, Adkison L, Bogaisky M, Carroll D, Carter E, Herlihy L, et al. Cost of Inpatient Falls and Cost-Benefit Analysis of Implementation of an Evidence-Based Fall Prevention Program. *JAMA Health Forum*. 2023;4:e225125. doi: https://dx.doi.org/10.1001/jamahealthforum.2022.5125
4. Yang Q, Li J, Shi D, Xie H, Wang J, Shi Z, Zhang Y. Incidence and risk factors associated with hospital-acquired pressure ulcers following total hip arthroplasty: A retrospective nationwide inpatient sample database study. *J Tissue Viability*. 2022;31:332-338. doi: 10.1016/j.jtv.2022.01.004
5. Chovanec K, Arsene C, Gomez C, Brixey M, Tolles D, Galliers JW, Kopaniasz R, Bobash T, Goodwin L. Association of CLABSI With Hospital Length of Stay, Readmission Rates, and Mortality: A Retrospective Review. *Worldviews Evid Based Nurs*. 2021;18:332-338. doi: 10.1111/wvn.12548
6. Jewell VD, Capistran K, Flecky K, Qi Y, Fellman S. Prediction of Falls in Acute Care Using The Morse Fall Risk Scale. *Occup Ther Health Care*. 2020;34:307-319. doi: 10.1080/07380577.2020.1815928
7. Haughey BS, White SC, Seckeler MD. Catheter-associated bloodstream infection incidence and outcomes in congenital cardiac surgery. *Congenit Heart Dis*. 2019;14:811-813. doi: 10.1111/chd.12809
8. Al-Qurayshi Z, Walsh J, Owen S, Kandil E. Surgical Site Infection in Head and Neck Surgery: A National Perspective. *Otolaryngol Head Neck Surg*. 2019;161:52-62. doi: 10.1177/0194599819832858
9. Gantz O, Zagadailov P, Merchant AM. The Cost of Surgical Site Infections after Colorectal Surgery in the United States from 2001 to 2012: A Longitudinal Analysis. *Am Surg*. 2019;85:142-149.
10. Hill AM, Jacques A, Chandler AM, Richey PA, Mion LC, Shorr RI. In-Hospital Sequelae of Injurious Falls in 24 Medical/Surgical Units in Four Hospitals in the United States. *Jt Comm J Qual Patient Saf*. 2019;45:91-97. doi: 10.1016/j.jcjq.2018.08.005
11. Fadayomi AB, Kasumova GG, Tabatabaie O, de Geus SWL, Kent TS, Ng SC, Moser AJ, Callery MP, Ashley SW, Tseng JF. Unique predictors and economic burden of superficial and deep/organ space surgical site infections following pancreatectomy. *HPB (Oxford)*. 2018;20:658-668. doi: 10.1016/j.hpb.2018.01.008
12. Sochet AA, Cartron AM, Nyhan A, Spaeder MC, Song X, Brown AT, Klugman D. Surgical Site Infection After Pediatric Cardiothoracic Surgery. *World J Pediatr Congenit Heart Surg*. 2017;8:7-12. doi: 10.1177/2150135116674467
13. Gonzalez DO, Ambeba E, Minneci PC, Deans KJ, Nwomeh BC. Surgical site infection after stoma closure in children: outcomes and predictors. *J Surg Res*. 2017;209:234-241. doi: 10.1016/j.jss.2016.10.029
14. Bauer K, Rock K, Nazzal M, Jones O, Qu W. Pressure Ulcers in the United States' Inpatient Population From 2008 to 2012: Results of a Retrospective Nationwide Study. *Ostomy Wound Manage*. 2016;62:30-38.
15. Brunelli SM, Turenne W, Sibbel S, Hunt A, Pfaffle A. Clinical and economic burden of bloodstream infections in critical care patients with central venous catheters. *J Crit Care*. 2016;35:69-74. doi: 10.1016/j.jcrc.2016.04.035

16. Mendizabal A, Thibault DP, Willis AW. Patient safety events in hospital care of individuals with epilepsy. *Epilepsia*. 2016;57:1301-1309. doi: 10.1111/epi.13440
17. Sears ED, Wu L, Waljee JF, Momoh AO, Zhong L, Chung KC. The Impact of Deep Sternal Wound Infection on Mortality and Resource Utilization: A Population-based Study. *World J Surg*. 2016;40:2673-2680. doi: 10.1007/s00268-016-3598-7
18. Dua A, Desai SS, Patel B, Seabrook GR, Brown KR, Lewis B, Rossi PJ, Malinowski M, Lee CJ. Preventable Complications Driving Rising Costs in Management of Patients with Critical Limb Ischemia. *Ann Vasc Surg*. 2016;33:144-148. doi: 10.1016/j.avsg.2015.11.026
19. McCutcheon BA, Ubl DS, Babu M, Maloney P, Murphy M, Kerezoudis P, Bydon M, Habermann EB, Parney I. Predictors of Surgical Site Infection Following Craniotomy for Intracranial Neoplasms: An Analysis of Prospectively Collected Data in the American College of Surgeons National Surgical Quality Improvement Program Database. *World Neurosurg*. 2016;88:350-358. doi: 10.1016/j.wneu.2015.12.068
20. Goudie A, Dynan L, Brady PW, Fieldston E, Brilli RJ, Walsh KE. Costs of Venous Thromboembolism, Catheter-Associated Urinary Tract Infection, and Pressure Ulcer. *Pediatrics*. 2015;136:432-439. doi: 10.1542/peds.2015-1386
21. Haider AH, Gupta S, Zogg CK, Kisat MT, Schupper A, Efron DT, Haut ER, Obirieze AC, Schneider EB, Pronvost PJ, et al. Beyond incidence: Costs of complications in trauma and what it means for those who pay. *Surgery*. 2015;158:96-103. doi: 10.1016/j.surg.2015.02.015
22. Deibert CM, Kates M, McKiernan JM, Spencer BA. National estimated costs of never events following radical prostatectomy. *Urol Oncol*. 2015;33:385 e381-386. doi: 10.1016/j.urolonc.2014.08.002
23. Allareddy V, Das A, Lee MK, Nalliah RP, Rampa S, Allareddy V, Rotta AT. Prevalence, predictors, and outcomes of methicillin-resistant Staphylococcus aureus infections in patients undergoing major surgical procedures in the United States: a population-based study. *Am J Surg*. 2015;210:59-67. doi: 10.1016/j.amjsurg.2014.08.041
24. Wilson MZ, Rafferty C, Deeter D, Comito MA, Hollenbeak CS. Attributable costs of central line-associated bloodstream infections in a pediatric hematology/oncology population. *Am J Infect Control*. 2014;42:1157-1160. doi: 10.1016/j.ajic.2014.07.025
25. Kandilov AM, Coomer NM, Dalton K. The impact of hospital-acquired conditions on Medicare program payments. *Medicare Medicaid Res Rev*. 2014;4. doi: 10.5600/mmrr.004.04.a01
26. Schweizer ML, Cullen JJ, Perencevich EN, Vaughan Sarrazin MS. Costs Associated With Surgical Site Infections in Veterans Affairs Hospitals. *JAMA Surg*. 2014;149:575-581. doi: 10.1001/jamasurg.2013.4663
27. Roy S, Patkar A, Daskiran M, Levine R, Hinoul P, Nigam S. Clinical and economic burden of surgical site infection in hysterectomy. *Surg Infect (Larchmt)*. 2014;15:266-273. doi: 10.1089/sur.2012.163
28. Goudie A, Dynan L, Brady PW, Rettiganti M. Attributable cost and length of stay for central line-associated bloodstream infections. *Pediatrics*. 2014;133:e1525-1532. doi: 10.1542/peds.2013-3795
29. Chiang HY, Kamath AS, Pottinger JM, Greenlee JD, Howard MA, 3rd, Cavanaugh JE, Herwaldt LA. Risk factors and outcomes associated with surgical site infections after craniotomy or craniectomy. *J Neurosurg*. 2014;120:509-521. doi: 10.3171/2013.9.JNS13843
30. Wang H, Niewczyk P, Divita M, Camicia M, Appelman J, Mix J, Sandel ME. Impact of pressure ulcers on outcomes in inpatient rehabilitation facilities. *Am J Phys Med Rehabil*. 2014;93:207-216. doi: 10.1097/PHM.0b013e3182a92b9c
31. Hsu E, Lin D, Evans SJ, Hamid KS, Frick KD, Yang T, Pronovost PJ, Pham JC. Doing well by doing good: assessing the cost savings of an intervention to reduce central line-associated bloodstream infections in a Hawaii hospital. *Am J Med Qual*. 2014;29:13-19. doi: 10.1177/1062860613486173

32. Shepard J, Ward W, Milstone A, Carlson T, Frederick J, Hadhazy E, Perl T. Financial impact of surgical site infections on hospitals: the hospital management perspective. *JAMA Surg*. 2013;148:907-914. doi: 10.1001/jamasurg.2013.2246
33. Bakkum-Gamez JN, Dowdy SC, Borah BJ, Haas LR, Mariani A, Martin JR, Weaver AL, McGree ME, Cliby WA, Podratz KC. Predictors and costs of surgical site infections in patients with endometrial cancer. *Gynecol Oncol*. 2013;130:100-106. doi: 10.1016/j.ygyno.2013.03.022
34. Liang MK, Li LT, Avellaneda A, Moffett JM, Hicks SC, Awad SS. Outcomes and predictors of incisional surgical site infection in stoma reversal. *JAMA Surg*. 2013;148:183-189. doi: 10.1001/jamasurgery.2013.411
35. Poultsides LA, Ma Y, Della Valle AG, Chiu YL, Sculco TP, Memtsoudis SG. In-hospital surgical site infections after primary hip and knee arthroplasty--incidence and risk factors. *J Arthroplasty*. 2013;28:385-389. doi: 10.1016/j.arth.2012.06.027
36. Harder EE, Gaies MG, Yu S, Donohue JE, Hanauer DA, Goldberg CS, Hirsch JC. Risk factors for surgical site infection in pediatric cardiac surgery patients undergoing delayed sternal closure. *J Thorac Cardiovasc Surg*. 2013;146:326-333. doi: 10.1016/j.jtcvs.2012.09.062
37. Memtsoudis SG, Dy CJ, Ma Y, Chiu YL, Della Valle AG, Mazumdar M. In-hospital patient falls after total joint arthroplasty: incidence, demographics, and risk factors in the United States. *J Arthroplasty*. 2012;27:823-828 e821. doi: 10.1016/j.arth.2011.10.010
38. Wong CA, Recktenwald AJ, Jones ML, Waterman BM, Bollini ML, Dunagan WC. The cost of serious fall-related injuries at three Midwestern hospitals. *Jt Comm J Qual Patient Saf*. 2011;37:81-87. doi: 10.1016/s1553-7250(11)37010-9
39. Carey K, Stefos T, Shibei Z, Borzecki AM, Rosen AK. Excess costs attributable to postoperative complications. *Med Care Res Rev*. 2011;68:490-503. doi: 10.1177/1077558710396378
40. Ho D, Lynch RJ, Ranney DN, Magar A, Kubus J, Englesbe MJ. Financial impact of surgical site infection after kidney transplantation: implications for quality improvement initiative design. *J Am Coll Surg*. 2010;211:99-104. doi: 10.1016/j.jamcollsurg.2010.02.055
41. Olsen MA, Butler AM, Willers DM, Gross GA, Hamilton BH, Fraser VJ. Attributable costs of surgical site infection and endometritis after low transverse cesarean delivery. *Infect Control Hosp Epidemiol*. 2010;31:276-282. doi: 10.1086/650755
42. de Lissovoy G, Fraeman K, Hutchins V, Murphy D, Song D, Vaughn BB. Surgical site infection: incidence and impact on hospital utilization and treatment costs. *Am J Infect Control*. 2009;37:387-397. doi: 10.1016/j.ajic.2008.12.010
43. Lee J, Singletary R, Schmader K, Anderson DJ, Bolognesi M, Kaye KS. Surgical site infection in the elderly following orthopaedic surgery. Risk factors and outcomes. *J Bone Joint Surg Am*. 2006;88:1705-1712. doi: 10.2106/JBJS.E.01156
44. Warren DK, Quadir WW, Hollenbeak CS, Elward AM, Cox MJ, Fraser VJ. Attributable cost of catheter-associated bloodstream infections among intensive care patients in a nonteaching hospital. *Crit Care Med*. 2006;34:2084-2089. doi: 10.1097/01.CCM.0000227648.15804.2D
45. McGarry SA, Engemann JJ, Schmader K, Sexton DJ, Kaye KS. Surgical-site infection due to Staphylococcus aureus among elderly patients: mortality, duration of hospitalization, and cost. *Infect Control Hosp Epidemiol*. 2004;25:461-467. doi: 10.1086/502422
46. Zhan C, Miller MR. Excess length of stay, charges, and mortality attributable to medical injuries during hospitalization. *JAMA*. 2003;290:1868-1874. doi: 10.1001/jama.290.14.1868
47. Dimick JB, Pelz RK, Consunji R, Swoboda SM, Hendrix CW, Lipsett PA. Increased resource use associated with catheter-related bloodstream infection in the surgical intensive care unit. *Arch Surg*. 2001;136:229-234. doi: 10.1001/archsurg.136.2.229
48. Yu KC, Jung M, Ai C. Characteristics, costs, and outcomes associated with central-line-associated bloodstream infection and hospital-onset bacteremia and fungemia in US hospitals. *Infect Control Hosp Epidemiol*. 2023;44:1920-1926. doi: 10.1017/ice.2023.132

49. Healy MA, Mullard AJ, Campbell DA, Jr., Dimick JB. Hospital and Payer Costs Associated With Surgical Complications. *JAMA Surg*. 2016;151:823-830. doi: 10.1001/jamasurg.2016.0773
50. Stroupe K, Manheim L, Evans C, Guihan M, Ho C, Li K, Cowper-Ripley D, Hogan T, St. Andre J, Huo Z, et al. Cost of Treating Pressure Ulcers for Veterans with Spinal Cord Injury. *Topics in Spinal Cord Injury Rehabilitation*. 2011;16:62-73. doi: 10.1310/sci1604-62

Supplemental Table 1: Study Characteristics

Author, year	Study design	Geographic location	Population description	Mean age	Female %	Setting	AE studied
Allareddy, 2015	Retrospective cohort	Nationwide	Patients selected from NIS during 2009-2010.	AE group: 56.5<br>No AE group: 47.8	AE group: 44<br>No AE group: 54	Multicenter	SSI
Al-Qurayshi, 2019	Cross-sectional	USA	Adult patients who underwent head and neck surgery as a primary admission procedure	AE group: 18-40: 18.5, >40 to <61: 43, 61 to <75: 22.9, ≥ 75: 15.6<br>No AE group: 18-40: 36.9, >40 to <61: 35.1, 61 to <75: 18.5, ≥ 75: 9.5	AE group: 36.4<br>No AE group: 51.6	Multicenter	SSI
Bakkum-Gomez, 2013	Retrospective cohort	Minnesota	Women who underwent Endometrial Cancer Screening between Jan 1999- December 2008	AE group: 63.8<br>No AE group: 64.3	NR	Academic	SSI
Bauer, 2016	Retrospective cohort	US Nationwide	Data from NIS between 2008 and 2012	71.2	1.6	Multicenter	Pressure ulcer
Brunelli, 2016	Retrospective cohort	Southern California and Nevada	Data from HealthCare Partners between January 1, 2011, and September 30,2014 of patients aged 18 years or older with a CVC placed during ICU stay	AE group: 70<br>No AE group: 70.7	AE group: 47.9<br>No AE group: 45.2	Multicenter	CLABSI
Carey, 2011	Retrospective cohort	US	Patients with postoperative complications from VA hospitals in the US	NR	NR	Multicenter	SSI
Chiang, 2014	Case-control	Iowa	Patients undergoing CRANI procedures at UIHC between 2006-2010 and developed SSI's	AE group: 52<br>No AE group: 52.5	NR	Academic	SSI
Chovanec, 2021	Retrospective cohort	USA	All hospitalized adult services patients in 2020 at any type of CVC at any point during an in-patient hospitalization.	AE group: 66<br>No AE group: 64	NR	Multicenter	CLABSI
de Lissovoy, 2009	Retrospective cohort	US Nationwide	Data from the 2005 HCUP NIS for 7 categories of surgical procedures	NR	NR	Multicenter	SSI

Author, year	Study design	Geographic location	Population description	Mean age	Female %	Setting	AE studied
			(neurological, cardiovascular, colorectal, skin, subcutaneous tissue, and breast; gastrointestinal, orthopedic, and obstetric and gynecologic)				
Deibert, 2015	Retrospective cohort	Nationwide	Males diagnosed with prostate cancer who were hospitalized to undergo radical prostatectomy.	NR	AE group: 0<br>No AE group: 0	Multicenter	Falls, pressure ulcer, CLABSI
Dimick, 2001	Prospective cohort	United States	Patients who stayed longer than 3 days at a large tertiary surgical ICU	NR	NR	Academic	CLABSI
Dua, 2016	Retrospective cohort	Nationwide	Patients with a primary diagnosis code of critical limb ischemia (CLI) were identified from the 2001-2011 in the NIS	NR	NR	Multicenter	SSI
Dykes, 2023	Case-control	New York, Boston	Patients who had a fall event with matched controls (no fall event)	AE group: <65: 47.3, ≥65: 52.7<br>No AE group: <65: 49, ≥65: 51	AE group: 46.6<br>No AE group: 46.2	Multicenter	Falls
Fadayomi, 2018	Retrospective cohort	US Nationwide	Data from ACS-NSQIP for adults ≥ 18 years who underwent elective pancreatectomies then suffered from superficial or deep /organ-space SSI	Superficial (65 years: 26.1; <65 years: 26.5); deep (65 years: 129.6; <65 years: 133.7)	NR	Multicenter	SSI
Gonzalez, 2016	Retrospective cohort	US Nationwide	Data from ACS-NSQIP Pediatric between 2012 and 2014 of children under 18 years undergoing surgical procedures	AE group: <28 weeks: 0; 29 weeks- 1 year: 79; 1-2 years: 21; 3-5 years: 12; 6-7 years: 6; 8-12 years: 13; 13-18 years: 30<br>No AE group: <28 weeks: 19; 29 weeks- 1 year: 1059; 1-2 years: 234; 3-5 years: 114; 6-7 years: 60;	AE group: 36<br>No AE group: 40.8	Multicenter	SSI

Author, year	Study design	Geographic location	Population description	Mean age	Female %	Setting	AE studied
				8-12 years: 146; 13-18 years: 317			
Goudie, 2014	Case-control	USA Nationwide	Data from NIS between 2008 and 2012 ages 18 years and younger	Neonate: 1336; <1 years old: 1002; 1-4 years old: 1916; 5-12 years: 1427; 13-17 years: 1027	42.41	Multicenter	CLABSI
Goudie, 2015	Retrospective cohort	Nationwide	At-risk patients from ages 1-17 years old selected from the NIS.	NR	NR	Multicenter	Pressure ulcer
Grantz, 2019	Retrospective cohort	USA Nationwide	Data from the NIS database from 2001 to 2012 of hospitalized patients who underwent elective and non-elective colorectal surgery and developed SSI, with matched controls to compare costs and LOS.	62.5	53.1	Multicenter	SSI
Haider, 2015	Retrospective cohort	Nationwide	Patients with specific AE diagnosis codes extracted from the NIS sample in 2008.	NR	NR	Multicenter	SSI
Harder, 2013	Case-control	Michigan	Patients who underwent delayed sternal closure between 2005 and 2009	AE group: 12 days<br>No AE group: 9 days	AE group: 53.5<br>No AE group: 42.6	Academic	SSI

Author, year	Study design	Geographic location	Population description	Mean age	Female %	Setting	AE studied
Haughey, 2019	Retrospective cohort	USA	inpatient admissions of patients ≤12 months old with ICD-9 codes for any diagnosis of congenital heart disease and a primary procedure code for congenital cardiac surgery	AE group: Neonates (<1 month): 0, Neonates (1-12 month): 4 months<br>No AE group: Neonates (<1 month): 0, Infants (1-12 months): 5 months	AE group: Neonates (<1 month): 46.4, Infants (1-12 months): 41.7<br>No AE group: Neonates (<1 month): 40.5, Infants (1-12 months): 47.8	Multicenter	CLABSI
Healy, 2016	Retrospective cohort	Michigan	Patients in the University of Michigan Health System from January 2, 2008, through April 16, 2015	NR	NR	Academic	SSI
Hill, 2019	Retrospective cohort	Tennessee	Patients who had a fall event with matched controls (two controls for each case)	AE group: 63.7<br>No AE group: 61.6	AE group: 50.6<br>No AE group: 59.7	Multicenter	Falls
Ho, 2010	Retrospective cohort	Michigan	Patients who underwent first time kidney transplant recipient	AE group: 53.4<br>No AE group: 48.6	NR	Academic	SSI
Hsu, 2014	Case-control	Hawaii	Patients aged 5 or older admitted to ICU from Jan 2009-Jan 2012 due to CLABSI	AE group: 55<br>No AE group: 61	AE group: 31<br>No AE group: 26	Academic	CLABSI
Jewell, 2020	Retrospective cohort	southwest USA	Adult patients, admitted as inpatient status, and fully admitted and evaluated by a nurse to receive an MFS assessment score.	AE group: 67.2<br>No AE group: 63.4	AE group: 41.1<br>No AE group: 54.4	Community	Falls
Kandilov, 2014	Retrospective cohort	Nationwide	PRESSURE ULCER: Medicare fee-for-service patients from 2008-2010 with select HACs<br>CLABSI: Medicare fee-for-service patients from 2008-2010 with select HACs<br>SSI: Medicare fee-for-service patients from 2008-2010 with select HACs	NR	PRESSURE ULCER: 54<br>CLABSI: 55<br>SSI: 58	Multicenter	Pressure ulcer, SSI, CLABSI

Author, year	Study design	Geographic location	Population description	Mean age	Female %	Setting	AE studied
Lee, 2006	Retrospective cohort	North Carolina and Virginia	Patients ages 64+ who underwent an orthopedic surgery in Duke University Medical Center and seven community hospitals in North Carolina and Virginia	AE group: 74.9<br>No AE group: 74.5	AE group: 66<br>No AE group: 66	Multicenter	SSI
Liang, 2013	Retrospective cohort	Michigan	Patients who underwent stoma reversal from January 1, 2005, until February 27, 2011.	AE group: 59<br>No AE group: 64	AE group: 5.4<br>No AE group: 6	Academic	SSI
Lu, 2023	Retrospective cohort	New York metropolitan area	Patients who were admitted for inpatient rehabilitation after acute hospitalization for COVID-19	AE group: 56.03<br>No AE group: 60.64	AE group: 17.9<br>No AE group: 34.6	Academic	Pressure ulcer
McCutcheon, 2016	Retrospective cohort	Nationwide	Patients who had one of the CPT codes but did not have an ASA physical status classification of 5 and had an operation for non-neoplastic reasons.	AE group: 53.69<br>No AE group: 56.23	AE group: 44.1<br>No AE group: 53.2	Multicenter	SSI
McGarry, 2014	Prospective cohort	North Carolina	Patients ages 70+ undergoing surgery from January 1, 1994, to November 30, 2000, at Duke University Medical Center and Durham Regional Hospital, focusing on with or without S. aureus infection	AE group: 75.5<br>No AE group: 75.8	AE group: 61.5<br>No AE group: 59.3	Multicenter	SSI
Memtsoudis, 2013	Retrospective cohort	US	Patients who underwent total joint arthroplasty between 1998 and 2007 from the National Inpatient Sample	68.2	AE group: 61.5<br>No AE group: 66.3	Multicenter	Falls
Mendizabal, 2016	Retrospective cohort	Nationwide	Data from NIS (2000–2010) ages 18 years or older were eligible for study and with an epilepsy diagnosis.	≤18	NR	Multicenter	Pressure ulcer, CLABSI

Author, year	Study design	Geographic location	Population description	Mean age	Female %	Setting	AE studied
Olsen, 2010	Retrospective cohort	Missouri	Patients who underwent low transverse cesarean section surgery	AE group: 26.7<br>No AE group: 27.2	NR	Community	SSI
Poultsides, 2013	Retrospective cohort	US	Patients who underwent Total knee Arthroplasty and Total Hip Arthroplasty between 1998 and 2007 from the National Inpatient Sample	AE group: 66<br>No AE group: 67	AE group: 55<br>No AE group: 61	Multicenter	SSI
Roy, 2014	Retrospective cohort	US Nationwide	Data from the Premier Perspectives Database for hysterectomies from January 2007-December 2009	Laparoscopic abdominal hysterectomy: 46.39; non-laparoscopic abdominal hysterectomy: 47.05; laparoscopic vaginal hysterectomy: 45.09; non-laparoscopic (entire population)	NR	Multicenter	SSI
Schweizer, 2014	Retrospective cohort	US Nationwide	Data from the VASQIP for non-Cardiac adults who received general, spinal, or epidural anesthesia between October 1, 2009, and September 30, 2010	AE group: 63.24<br>No AE group: 62.98	AE group: 5.47<br>No AE group: 6.11	Multicenter	SSI
Sears, 2016	Retrospective cohort	Nationwide	All patients aged 18 years or older who underwent open cardiac surgery during the study period from 2009-2013.	AE group: 62<br>No AE group: 63	AE group: 34<br>No AE group: 28	Multicenter	SSI
Shepard, 2013	Retrospective cohort	Maryland	Inpatients who developed an SSI after certain surgical procedures from January 2007-December 2010	NR	NR	Multicenter	SSI
Sochet, 2016	Retrospective cohort	Washington, DC	Children aged 0 to 18 years with SSI who were admitted to CICU post CTS from January 2010 through December 2013, with matched controls	AE group: 8.9 months<br>No AE group: 9.6 months	NR	Academic	SSI

Author, year	Study design	Geographic location	Population description	Mean age	Female %	Setting	AE studied
Stroupe, 2011	Retrospective cohort	United States	Patients who were diagnosed with a spinal cord injury/disorder	AE group: 59.4<br>No AE group: 59.4	AE group: 2.1<br>No AE group: 2.9	Academic	Pressure ulcer
Wang, 2014	Retrospective cohort	US Nationwide	Data from the IRF for adults greater than 18 years old between 2009-2011	AE group: 74.9<br>No AE group: 74.9	AE group: 69.1<br>No AE group: 69.1	Multicenter	Pressure ulcer
Warren, 2006	Prospective cohort	Missouri	All patients admitted from January 19, 1998, to July 31, 2000, in a ten-bed medical ICU and ten-bed surgical ICU of Missouri Baptist Medical Center	AE group: 70<br>No AE group: 71	AE group: 44<br>No AE group: 47	Community	CLABSI
Wilson, 2014	Retrospective cohort	Hershey, Pennsylvania	Patients admitted as inpatients to the pediatric HEM/ONC service between 2008-2011.	AE group: 7.5<br>No AE group: 9	AE group: 30<br>No AE group: 34.89	Academic	CLABSI
Wong, 2011	Case-control	Missouri	Patients who experienced serious fall-related injuries in three hospitals in Missouri	AE group: 68.8<br>No AE group: 68.9	AE group: 54.4<br>No AE group: 57.8	Community	Falls
Yang, 2022	Retrospective cohort	USA Nationwide	Patients' data was extracted from NIS database from 2005 to 2014. The recruited cases were divided into two groups according to the occurrence of hospital-acquired pressure ulcers.	AE group: 70<br>No AE group: 66	AE group: 52.09<br>No AE group: 56.07	Multicenter	Pressure ulcer
Yin, 2023	Retrospective cohort	Texas	Hospital discharge data of patients aged ≥60 years diagnosed with and without sacral ulcers from April to September 2021	AE group: 76.5<br>No AE group: 74.3	AE group: 52<br>No AE group: 55	Multicenter	Pressure ulcer
Yu, 2023	Retrospective cohort	United States	Patients in 41 acute-care hospitals in the BD Insights Research and Database	NR	AE group: 47.9<br>No AE group: 42.9	Multicenter	CLABSI
Zhan, 2003	Retrospective cohort	US Nationwide	Data from 2000 NIS of all inpatient stays of 994 acute-care general hospitals across 28 states	NR	NR	Multicenter	Pressure ulcer

Supplemental Table 2: Methodological Quality

Author, year	Representativeness of cohort 1	Representativeness of cohort 2	Ascertainment of exposure	Outcome absence at study start	Comparability	Ascertainment of outcome: independent blind assessment	Follow-up long enough for outcomes to occur	Adequacy of follow-up
Allareddy, 2015	Low risk	Low risk	Low risk	Low risk	High risk	Low risk	Low risk	Low risk
Al-Qurayshi, 2019	Low risk	Low risk	Low risk	Low risk	High risk	Low risk	Low risk	Low risk
Bakkum-Gomez, 2013	Low risk	Low risk	Low risk	Low risk	High risk	Low risk	Low risk	Low risk
Bauer, 2016	Low risk	Low risk	Low risk	Low risk	High risk	Low risk	Low risk	Low risk
Brunelli, 2016	Low risk	Low risk	Low risk	Low risk	High risk	Low risk	Low risk	Low risk
Carey, 2011	Low risk	Low risk	Low risk	Low risk	No information	Low risk	Low risk	Low risk
Chiang, 2014	Low risk	Low risk	Low risk	Low risk	High risk	Low risk	Low risk	Low risk
Chovanec, 2021	Low risk	Low risk	Low risk	Low risk	High risk	Low risk	Low risk	Low risk
de Lissovoy, 2009	Low risk	Low risk	Low risk	Low risk	Low risk	Low risk	Low risk	Low risk
Deibert, 2015	Low risk	Low risk	Low risk	Low risk	High risk	Low risk	Low risk	Low risk
Dimick, 2001	Low risk	Low risk	Low risk	Low risk	No information	Low risk	Low risk	Low risk
Dua, 2016	Low risk	Low risk	Low risk	Low risk	No information	Low risk	Low risk	Low risk
Dykes, 2023	Low risk	Low risk	Low risk	Low risk	Low risk	Low risk	Low risk	Low risk
Fadayomi, 2018	Low risk	Low risk	Low risk	Low risk	High risk	Low risk	Low risk	Low risk
Gonzalez, 2016	Low risk	Low risk	Low risk	Low risk	Low risk	Low risk	Low risk	Low risk
Goudie, 2014	Low risk	Low risk	Low risk	Low risk	High risk	Low risk	Low risk	Low risk
Goudie, 2015	Low risk	Low risk	Low risk	Low risk	High risk	Low risk	Low risk	Low risk
Grantz, 2019	Low risk	Low risk	Low risk	Low risk	High risk	Low risk	Low risk	Low risk
Haider, 2015	Low risk	Low risk	Low risk	Low risk	High risk	Low risk	Low risk	Low risk
Harder, 2013	Low risk	Low risk	Low risk	Low risk	High risk	Low risk	Low risk	Low risk
Haughey, 2019	Low risk	Low risk	Low risk	Low risk	High risk	Low risk	Low risk	Low risk
Healy, 2016	Low risk	Low risk	Low risk	Low risk	High risk	Low risk	Low risk	Low risk
Hill, 2019	Low risk	Low risk	Low risk	Low risk	High risk	Low risk	Low risk	Low risk

Author, year	Representativeness of cohort 1	Representativeness of cohort 2	Ascertainment of exposure	Outcome absence at study start	Comparability	Ascertainment of outcome: independent blind assessment	Follow-up long enough for outcomes to occur	Adequacy of follow-up
Ho, 2010	Low risk	Low risk	Low risk	Low risk	High risk	Low risk	Low risk	Low risk
Hsu, 2014	Low risk	Low risk	Low risk	Low risk	Low risk	Low risk	Low risk	Low risk
Jewell, 2020	Low risk	Low risk	Low risk	Low risk	High risk	Low risk	Low risk	Low risk
Kandilov, 2014	Low risk	Low risk	Low risk	Low risk	No information	Low risk	Low risk	Low risk
Lee, 2006	Low risk	Low risk	Low risk	Low risk	Low risk	Low risk	Low risk	Low risk
Liang, 2013	Low risk	Low risk	Low risk	Low risk	High risk	Low risk	Low risk	Low risk
Lu, 2023	Low risk	Low risk	Low risk	Low risk	Low risk	Low risk	Low risk	Low risk
McCutcheon, 2016	Low risk	Low risk	Low risk	Low risk	High risk	Low risk	Low risk	Low risk
McGarry, 2014	Low risk	Low risk	Low risk	Low risk	Low risk	Low risk	Low risk	Low risk
Memtsoudis, 2013	Low risk	Low risk	Low risk	Low risk	High risk	Low risk	Low risk	Low risk
Mendizabal, 2016	Low risk	Low risk	Low risk	Low risk	No information	Low risk	Low risk	Low risk
Olsen, 2010	Low risk	Low risk	Low risk	Low risk	High risk	Low risk	Low risk	Low risk
Poultsides, 2015	Low risk	Low risk	Low risk	Low risk	High risk	Low risk	Low risk	Low risk
Roy, 2014	Low risk	Low risk	Low risk	Low risk	Low risk	Low risk	Low risk	Low risk
Schweizer, 2014	Low risk	Low risk	Low risk	Low risk	High risk	Low risk	Low risk	Low risk
Sears, 2016	Low risk	Low risk	Low risk	Low risk	High risk	Low risk	Low risk	Low risk
Shepard, 2013	Low risk	Low risk	Low risk	Low risk	No information	Low risk	Low risk	Low risk
Sochet, 2016	Low risk	Low risk	Low risk	Low risk	Low risk	Low risk	Low risk	Low risk
Stroupe, 2011	Low risk	Low risk	Low risk	Low risk	High risk	Low risk	Low risk	Low risk
Wang, 2014	Low risk	Low risk	Low risk	Low risk	High risk	Low risk	Low risk	Low risk
Warren, 2006	Low risk	Low risk	Low risk	Low risk	High risk	Low risk	Low risk	Low risk
Wilson, 2014	Low risk	Low risk	Low risk	Low risk	High risk	Low risk	Low risk	Low risk
Wong, 2011	Low risk	Low risk	Low risk	Low risk	High risk	Low risk	Low risk	Low risk
Yang, 2022	Low risk	Low risk	Low risk	Low risk	Low risk	Low risk	Low risk	Low risk
Yin, 2023	Low risk	Low risk	Low risk	Low risk	Low risk	Low risk	Low risk	Low risk

Author, year	Representativeness of cohort 1	Representativeness of cohort 2	Ascertainment of exposure	Outcome absence at study start	Comparability	Ascertainment of outcome: independent blind assessment	Follow-up long enough for outcomes to occur	Adequacy of follow-up
Yu, 2023	Low risk	Low risk	Low risk	Low risk	No information	Low risk	Low risk	Low risk
Zhan, 2003	Low risk	Low risk	Low risk	Low risk	No information	Low risk	Low risk	Low risk

Supplemental Table 3: Comparison of cost differences (in USD) before and after HACRP Implementation (2000-2024)

HAC	2000 – 2024 studies			2000-2013 studies, pre-HACRP			2014-2024 studies, post-HACRP			Difference in mean value
	No. studies	No. patients	Mean excess value	No. studies	No. patients	Mean excess value	No. studies	No. patients	Mean excess value	
**Falls**	9	1,184,802	23,686	1	166	8,350	8	1,184,636	27,520	19,170↑
**Pressure ulcer**	6	4,639,644	33,942	1	1,220	77,587	5	673,137	25,213	52,374↓
**CLABSI**	12	94,802	104,349	3	1,692	67,945	9	45,709	117,999	50,054↑
**SSI**	33	25,432,527	23,727	21	1,932,656	17,217	12	23,499,357	30,217	13,000↑

Cost

For falls, healthcare costs per patient increased significantly, rising from $8,350 pre-HACRP to $27,520 post-HACRP, marking a $19,170 increase. Pressure ulcers saw a notable decrease in costs, with a drop from $77,587 pre-HACRP to $25,213 post-HACRP, resulting in a $52,374 reduction in costs. In contrast, CLABSI costs surged, rising by $50,054 from $67,945 pre-HACRP to $117,999 post-HACRP. Lastly, for SSI, the cost per patient increased by $13,000, from $17,217 pre-HACRP to $30,217 post-HACRP

Supplemental Table 4: PRISMA Check List

Section/topic	#	Checklist item	Reported on page #
**TITLE**			
Title	1	Identify the report as a systematic review, meta-analysis, or both.	1
**ABSTRACT**			
Structured summary	2	Provide a structured summary including, as applicable: background; objectives; data sources; study eligibility criteria, participants, and interventions; study appraisal and synthesis methods; results; limitations; conclusions and implications of key findings; systematic review registration number.	2
**INTRODUCTION**			
Rationale	3	Describe the rationale for the review in the context of what is already known.	4
Objectives	4	Provide an explicit statement of questions being addressed with reference to participants, interventions, comparisons, outcomes, and study design (PICOS).	6
**METHODS**			
Protocol and registration	5	Indicate if a review protocol exists, if and where it can be accessed (e.g., Web address), and, if available, provide registration information including registration number.	NA
Eligibility criteria	6	Specify study characteristics (e.g., PICOS, length of follow-up) and report characteristics (e.g., years considered, language, publication status) used as criteria for eligibility, giving rationale.	6
Information sources	7	Describe all information sources (e.g., databases with dates of coverage, contact with study authors to identify additional studies) in the search and date last searched.	6-7
Search	8	Present full electronic search strategy for at least one database, including any limits used, such that it could be repeated.	7
Study selection	9	State the process for selecting studies (i.e., screening, eligibility, included in systematic review, and, if applicable, included in the meta-analysis).	6
Data collection process	10	Describe method of data extraction from reports (e.g., piloted forms, independently, in duplicate) and any processes for obtaining and confirming data from investigators.	6

Data items	11	List and define all variables for which data were sought (e.g., PICOS, funding sources) and any assumptions and simplifications made.	6-8
Risk of bias in individual studies	12	Describe methods used for assessing risk of bias of individual studies (including specification of whether this was done at the study or outcome level), and how this information is to be used in any data synthesis.	7
Summary measures	13	State the principal summary measures (e.g., risk ratio, difference in means).	8-9
Synthesis of results	14	Describe the methods of handling data and combining results of studies, if done, including measures of consistency (e.g., I^2) for each meta-analysis.	NA
Section/topic	#	Checklist item	Reported on page #
Risk of bias across studies	15	Specify any assessment of risk of bias that may affect the cumulative evidence (e.g., publication bias, selective reporting within studies).	8-9
Additional analyses	16	Describe methods of additional analyses (e.g., sensitivity or subgroup analyses, meta-regression), if done, indicating which were pre-specified.	NA
**RESULTS**			
Study selection	17	Give numbers of studies screened, assessed for eligibility, and included in the review, with reasons for exclusions at each stage, ideally with a flow diagram.	8-9
Study characteristics	18	For each study, present characteristics for which data were extracted (e.g., study size, PICOS, follow-up period) and provide the citations.	Suppl
Risk of bias within studies	19	Present data on risk of bias of each study and, if available, any outcome level assessment (see item 12).	Suppl
Results of individual studies	20	For all outcomes considered (benefits or harms), present, for each study: (a) simple summary data for each intervention group (b) effect estimates and confidence intervals, ideally with a forest plot.	Suppl
Synthesis of results	21	Present results of each meta-analysis done, including confidence intervals and measures of consistency.	NA
Risk of bias across studies	22	Present results of any assessment of risk of bias across studies (see Item 15).	Suppl
Additional analysis	23	Give results of additional analyses, if done (e.g., sensitivity or subgroup analyses, meta-regression [see Item 16]).	NA
**DISCUSSION**			
Summary of evidence	24	Summarize the main findings including the strength of evidence for each main outcome; consider their relevance to key groups (e.g., healthcare providers, users, and policy makers).	12

Limitations	25	Discuss limitations at study and outcome level (e.g., risk of bias), and at review-level (e.g., incomplete retrieval of identified research, reporting bias).	14-15
Conclusions	26	Provide a general interpretation of the results in the context of other evidence, and implications for future research.	
**FUNDING**			
Funding	27	Describe sources of funding for the systematic review and other support (e.g., supply of data); role of funders for the systematic review.	
